# Carbapenem Therapeutic Drug Monitoring in Critically Ill Adult Patients and Clinical Outcomes: A Systematic Review with Meta-Analysis

**DOI:** 10.3390/antibiotics10020177

**Published:** 2021-02-10

**Authors:** Sharon Lechtig-Wasserman, Hans Liebisch-Rey, Nicolas Diaz-Pinilla, Jhosep Blanco, Yuli-Viviana Fuentes-Barreiro, Rosa-Helena Bustos

**Affiliations:** Evidence-Based Therapeutics Group, Clinical Pharmacology, Universidad de La Sabana, Chía 140013, Colombia; sharonlele@unisabana.edu.co (S.L.-W.); hanslire@unisabana.edu.co (H.L.-R.); nicolasdipi@unisabana.edu.co (N.D.-P.); jhosepblme@unisabana.edu.co (J.B.); yulyfuba@unisabana.edu.co (Y.-V.F.-B.)

**Keywords:** critical illness, septic shock, sepsis, carbapenems, therapeutic drug monitoring, antibiotic treatment outcome, antimicrobial drug resistance, gram-negative bacteria

## Abstract

Drug monitoring is one strategy of antibiotic stewardship to face antimicrobial resistance. This strategy could have a determinant role in critically ill patients treated with carbapenems to overcome pharmacokinetic variability, reduce the risk of subtherapeutic dosage or toxicity, and reduce the risks inherent to treatment. However, the effectiveness of therapeutic drug monitoring (TDM) is unknown. This paper aims to identify TDM effectiveness in critically ill patients treated with carbapenems. English and ClinicalTrials.gov databases were searched to identify relevant studies evaluating carbapenem TDM. Randomized controlled trials (RCTs) and comparative cohort studies were selected for inclusion if they compared carbapenem TDM to standard care in adult critically ill or sepsis/septic shock patients. The primary outcome was mortality. Secondary outcomes included morbidity, clinical cure, microbiological eradication, antimicrobial resistance, drug-related side effects, and achievement of target plasma concentrations. Overall, performing carbapenem TDM was not associated with a decrease in mortality. However, it could be evidence for a relationship with clinical cure as well as target attainment. Some studies found favorable outcomes related to clinical and microbiological responses, such as lower procalcitonin levels at the end of the monitored therapy compared to standard care. For the primary and secondary outcomes analyzed, strong evidence was not identified, which could be due to the size, risk of bias, and design of selected studies.

## 1. Introduction

In the treatment of infectious diseases, the therapeutic index (TI) of antimicrobials can show significant pharmacokinetic (PK) variability. It is of great importance to know the concentration of the antibiotic, which must be higher than the minimum effective concentration (MEC) for a favorable clinical outcome for the patient [1] to avoid antibiotic-resistant bacteria [2]. The measurement of the concentration of drugs in fluids such as plasma, serum, or blood in patients at specific intervals is called therapeutic drug monitoring (TDM) [3,4]. PK (study of drug processes throughout the body, such as absorption, distribution, metabolism, and excretion) and pharmacodynamic (PD) (drug transformation processes at the site of action and its pharmacological effect) can vary [5] to adapt the drug dose and improve efficacy and/or reduce toxicity. The measurement of drug concentrations allows the dose to be adjusted to an adequate therapeutic range [6]. There are several indications to perform TDM, such as drugs with a narrow therapeutic index (NTI), high variability or unpredictability between drug dose and plasma concentration, situations where there is knowledge of the clinical effect and/or toxicity related to concentration, adverse effects defined by overdose or insufficient dose, and availability of equipment and personnel for the processing and interpretation of the results of TDM [1,7,8]. NTI is used to identify those drugs where small differences in dose or blood concentration may lead to dependence, therapeutic failure, or side effects. It has also been proven that there is substantial intra and inter-individual PK variability between patients, especially those with critical illnesses [9]. According to the World Health Organization (WHO), drugs that require TDM are anticonvulsants, antiarrhythmics, immunosuppressants, and antibiotics (fluoroquinolones, lipopeptides, glycopeptides, and β-lactams, among many others) [4].

Carbapenems therapeutic drug monitoring is of particular interest due to its extended-spectrum activity against Gram-negative, Gram-positive and β-lactamases producing microorganisms, and their role in the treatment of severe infections [10]. Empirical monotherapy of carbapenems for serious infections has been reported in the literature as safe and effective [11] and as second-line therapy when the first is insufficient [12]. The bactericidal activity of carbapenems can be compromised due to different mechanisms of intrinsic resistance (insensitivity), acquired enzymatic inactivation such as target-site mutation and efflux pumps, or both. Some studies have found alterations of porin channels in the cell membrane of Gram-negative bacteria as a possible mechanism of resistance [13]. On the other hand, B-lactamases with carbapenemase activity (collectively called car-bapenemases), which can be class A (e.g., Klebsiella pneumoniae carbapenemase, KPC), class D (e.g., OXA-48) and all class B Metallo-B-lactamases (e.g., NDM-1), confer resistance to carbapenems [14]. The presence of these enzymes limits the role of carbapenems in the treatment of severe infections [15].

The parameters that correlate the efficacy of carbapenems are area under the curve (AUC) to the ratio of the minimum inhibitory concentration (MIC), exposure time over MIC (*fT_>MIC_* expressed as a percentage of the administration interval) and the maximum concentration concerning the MIC ratio [11]. Carbapenems, being time-dependent, allow increasing efficacy by extending the infusion time without the need to increase the dose [16]. Carbapenems require a low percentage of T_>MIC_ and have a beneficial post-antibiotic effect [17]. These types of antibiotics quickly penetrate different tissues and interstitial fluid with a penetration rate of 20% [18,19]. Due to its extracellular distribution, the volume of distribution (Vd) is between 15 to 20 L [19]. The elimination is mainly renal; thus, dose adjustments are necessary in patients with renal failure. The elimination half-life is around 1 h for most carbapenems, however, for ertapenem, it is around 3.8 h [18].

On the other hand, the definition of a critically ill patient is broad and may not be clear in the available scientific literature. Nonetheless, the American College of Critical Care, Society of Critical Care Medicine includes within the group of critically ill patients the following types of patients: unstable patients (acute respiratory dysfunction requiring ventilatory support and patients with shock or unstable hemodynamic state that require invasive monitoring or vasoactive drugs); patients needing intensive follow-up and immediate treatment that cannot be provided outside the ICU; an acute disease with a high risk of deterioration or death requiring monitoring and medical or surgical interventions [20]. Microorganisms displaying higher MICs are frequently isolated from critically ill patients. This is because PK variability is enhanced, and as such, dose adjustments must be made carefully, taking special care with clinical breakpoints or surrogate MICs [21]. In this sense, the use of TDM plays an important role in avoiding antimicrobial resistance and allowing a favorable clinical outcome.

As carbapenems are time-dependent, T_>MIC_ takes on greater relevance than an adequate concentration [22]. Reports have shown that in critically ill patients, the best clinical results have been when the *fT_>MIC_* is 75–100%. However, it is not fully understood whether reaching *fT_>4MIC_* leads to better clinical outcomes [23]. Regarding bacteriostatic effects, an *fT_>MIC_* of approximately20% is sufficient, and for achieving bactericidal effects, and *fT_>MIC_* of approximately 40% is necessary [17].

In line with the above-mentioned, the alteration of PK parameters has implications in the clinic, as does the administration of an adequate dose of antibiotics, making a case for the implementation of routine TDM [24]. However, the practice and benefits of carbapenem TDM in critically ill patients is not clear [25]. The aim of this review is to identify the efficacy of TDM in these types of patients in terms of clinical and bioanalysis results, and as a strategy to reduce antimicrobial resistance.

## 2. Results

### 2.1. Search Results

**Figure 1:**
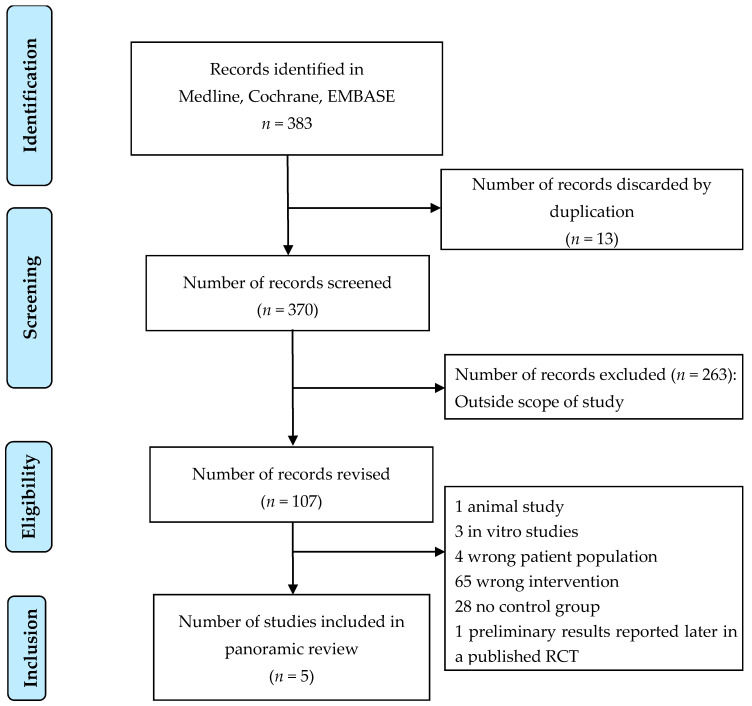
Flowchart of the search strategy and study selection.

The reviewers screened 383 articles selected from electronic databases according to the search criteria described in Table S1. After removing duplicates and articles outside the scope of the study, 107 studies were eligible based on the assessment of titles and abstracts. A further 102 studies were excluded based on the review of full-text articles because of their population, intervention, or the absence of a control group. One study was also excluded because it contained preliminary results of a randomized controlled trial (RCTs) published later. Five studies were included, two of them were RCTs and three were retrospective cohort studies (Figure 1).

### 2.2. Study Characteristics

Five studies were included according to their study design and relation with the aims of this study. Two of the studies were single-center RCTs and the other three were single-center retrospective cohort studies. All selected studies included meropenem as the main carbapenem of interest, however, Fournier also included imipenem and ertapenem [26]. The studies were partially blinded and used chromatography analysis to establish antibiotic plasma concentrations. De Waele’s RCT had a unique combined antibiotic exposure (meropenem and piperacillin/tazobactam) without availability of reliable absolute frequencies, therefore, it was not considered for the meta-analysis [27].

**Figure 2:**
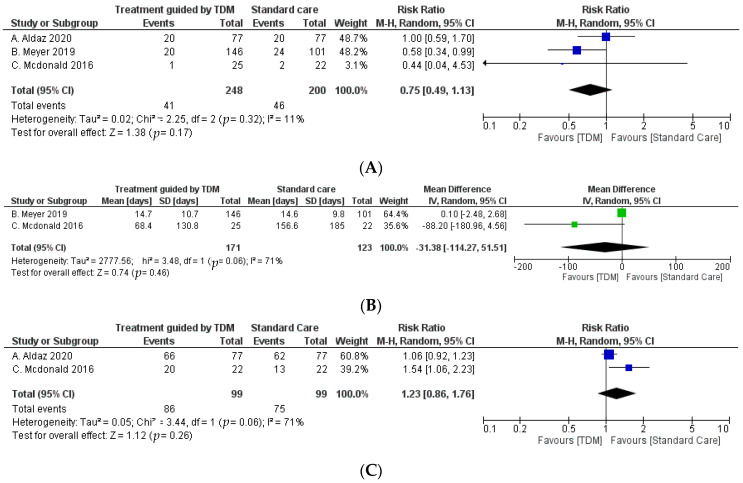
Forest plots. Therapeutic drug monitoring (TDM) cohort versus standard care for in-hospital mortality (observational) (**A**). TDM cohort versus standard care for microbiological eradication (observational) (**B**). TDM cohort versus standard care for intensive care unit (ICU) length of stay in days (observational) (**C**).

For the primary outcome, a total of four out of five studies included mortality, although just three of them (observational studies) were included to perform the meta-analysis, with a total of 448 patients, with 248 assigned to the treatment guided by TDM and 200 assigned to the standard care group [28,29,30]. Only two of the secondary outcomes from observational studies could be performed with meta-analysis, which were ICU length of stay and microbiological eradication, with a total of 294 and 198 patients, respectively (Figure 2). For the rest of the secondary outcomes, a descriptive summary was performed because of their intervention/exposition variability or high risk of bias. The description of studies selected, and clinical outcomes are shown in Table 1 and Table 2 respectively.

### 2.3. Clinical Outcome

For combined effect in the primary clinical outcome (in-hospital mortality), 41 of the 248 patients (16.53%) died in the TDM cohort and 46 of 200 patients (23%) died in the standard care group. The pooled relative risk of in-hospital mortality did not show a statistically significant difference between the groups (0.75 [95% confidence interval 0.49 to 1.13]), although Meyer’s research showed differences that favor TDM (Figure 2) [29]. In the secondary clinical outcomes, the pooled relative risk of the ICU length of stay and microbiological eradication between groups were statistically non-significant with a mean difference of −31.38 [95% confidence interval −180.96 to 4.56] and a relative risk of 1.23 [95% confidence interval 0.86 to 1.76], respectively (Figure 2). For the rest of secondary outcomes only a systematic review was performed (Table 2).

As for achieving clinical cure, Aldaz et al. found a statistically significant reduction in procalcitonin (at least 80%) in the TDM cohort compared with the control (71% versus 53%, *p*-value equal to 0.02) [28]. For target attainment, studies were heterogenous in their measurements of pharmacokinetic parameters, however, both De Waele et al. and McDonald et al. measured 100% *fT_>MIC_*, with a statistically significant higher attainment of target levels in the TDM group in the former (94.7% versus 68.4% with *p*-value of 0.045), which also reported a statistically significant difference in the 100% *fT_>4MIC_* between groups (57.9% versus 15.8% with *p*-value equal to 0.007) [27,30].

De Waele et al. and Aldaz et al. included antimicrobial persistence (antimicrobial resistance was not measured in any study), with statistically non-significant differences between groups [27,28]. Adverse reactions were another evaluated outcome, with Aldaz and McDonald observing no significant differences between groups for various systems and organs [28,30]. Finally, hospital re-admission was evaluated in a cohort study (Aldaz) which found a small, but non-significant reduction in the patients re-admitted in the group that received therapeutic drug monitoring, compared to the control group (6.49% versus 9.09% with *p*-value of 0.54) [28].

### 2.4. Heterogeneity of Studies

For the primary clinical outcome (in-hospital mortality), heterogeneity among studies was not observed (I^2^ = 11%; χ^2^ = 2.25; *p*-value = 0.32) (Figure 2). The heterogeneity for secondary outcomes (ICU length of stay and microbiological eradication) is described in each forest plot (Figure 2).

### 2.5. Publication Bias, Risk of Bias, and Quality of Evidence

**Figure 3:**
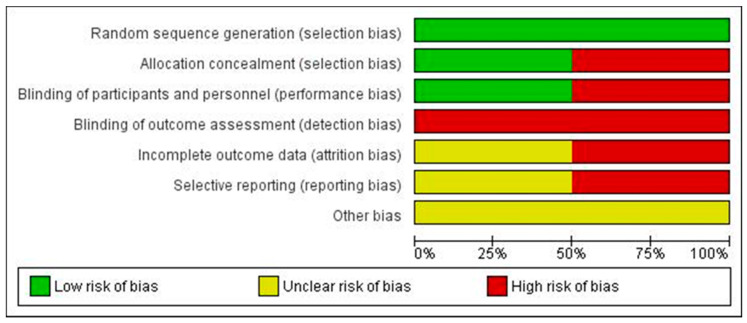
Risk of bias in randomized controlled trials using Cochrane risk of bias tool.

**Figure 4:**
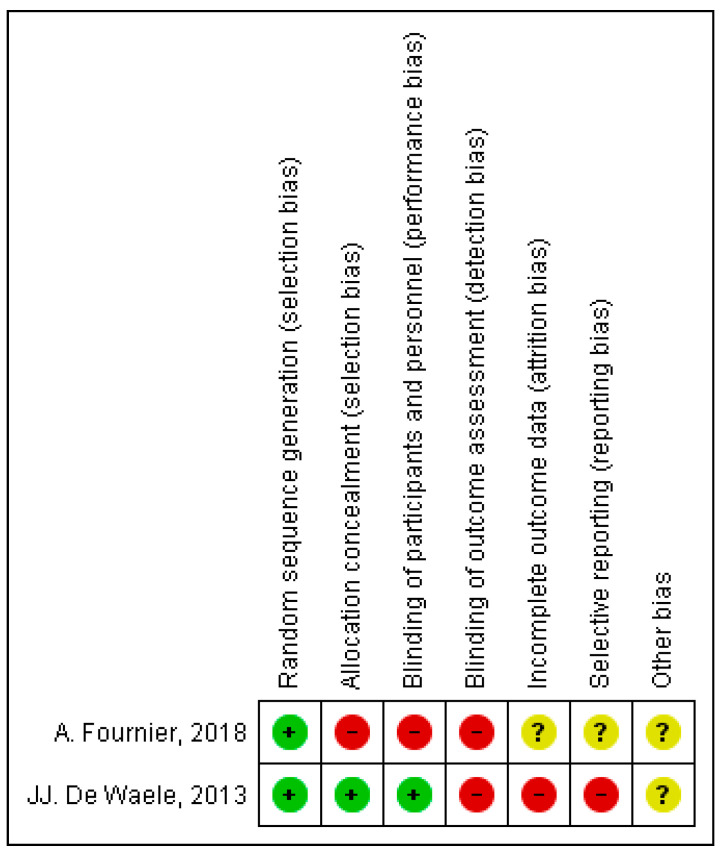
Summary of risk of bias in selected studies (Cochrane Risk of Bias Tool for randomized controlled trials).

We did not test for the presence of publication bias for any outcome because there were less than 10 studies. For the selected RCTs we found a high risk of bias, mainly in the blinding processes, incomplete outcome data and selective reporting of information (Figure 3 and Figure 4) [26,27]. For observational studies, Aldaz’s had a low risk of bias and the remaining studies received a moderate risk of bias score, with blinding mechanisms and follow-up considered the major problems (Table 3) [28,29,30].

## 3. Discussion

To our knowledge, this is the first systematic review and meta-analysis to assess the clinical outcomes of performing carbapenem TDM. TDM is an underused practice and its application has been limited to a few antibiotics with PK features that increase the risk of clinical failure and toxicity, like vancomycin and aminoglycosides [31,32]. β-Lactam and, specifically, carbapenem TDM have not been widely investigated because of the wide TI associated with these antibiotics. However, some populations would benefit from performing TDM because of their intra and interindividual PK variability; the unpredictable PK in critically ill patients is the main reason why TDM could optimize antibiotic treatment, for β-lactams and especially carbapenems despite their wide TI, leading either to reduced antibiotic resistance and adverse effects or enhanced clinical or microbiological cure. Although, evidence of the relationship between implementation of TDM and improved outcomes is, according to our findings, substantially less solid.

For the critically ill population in particular, optimizing therapy ensuring an adequate antibiotic exposure can be challenging, because sepsis itself can induce multiple organ dysfunction that leads to a reduction of antibiotic clearance [33], increasing the likelihood of toxicity [33] and reducing the exposure, making it more difficult to achieve the PK/PD target. According to Blot et al., some other hemodynamic changes that occur in critically ill patients which alter PD are homeostatic disturbance, endothelial dysfunction, capillary leak, decreased plasma protein concentrations, and extreme body weight changes [34]. Some PK changes in this population include increased Vd, augmented renal clearance, hypoalbuminemia (which alters the unbound fraction of the drug), and reduced bacterial susceptibility [35]. Therefore, prescription of standard doses is likely to result in sub-therapeutic concentrations [34], increasing the likelihood of therapeutic failure.

Furthermore, dangerous adverse effects resulting from antibiotic use could be avoided with the use of TDM [3], particularly, by maintaining plasma levels below the threshold of the minimum toxic concentration (MTC), also called maximum safe concentration (MSC), which is when unacceptable adverse effects begin to take place [36]. In the case of carbapenems, neurotoxicity—chiefly in the form of seizures—is particularly concerning when patients are overdosed relative to weight or renal function [37].

We selected mortality as our primary outcome. As shown in Table 2, TDM was not associated with inferior mortality rates. The lack of efficacy of TDM for improving mortality rates may relate to some inconsistencies found in the selected studies, including study design, number of participants, procedures, and methodological disparities. Only the study by Meyer et al. had statistically significant results that support this outcome [29]. It is likely that new experiments as well as bigger studies could show major differences. Because meropenem has a post-antibiotic effect, prolonged follow-up durations may be necessary to detect TDM-related benefits in terms of mortality. However, most of the included studies, including both RCTs, did not assess outcomes after hospital discharge, and there are no studies that measure mortality rates in a significant period.

According to the meta-analysis performed in this review, TDM does not significantly affect the ICU length of stay of patients. This result could be due to the design of the studies and the size of both the TDM cohort and control groups in the studies analyzed. However, a multi-center RCT, the DOLPHIN trial, is underway, designed to assess the efficacy and cost-effectiveness of model-based TDM of β-lactam and fluoroquinolones, their primary outcome is the ICU length of stay [38]. This systematic review is intended to be updated when the DOlPHIN trial shows its first results. On the other hand, microbiological eradication and TDM did not show a direct relationship in our study. However, studies such as those by Bricheux et al. have found failure occurred in a large proportion of patients whose dose was not increased, though the difference between the groups were not statistically significant [39].

The results shown in this review are not sufficient to demonstrate that meropenem TDM helps reach a defined PK/PD target. The only study that had statistically significant results regarding performing TDM and antimicrobial exposure was the RCT by De Waele et al., which had important biases [27]. Some studies demonstrate that reaching PK/PD targets is related to better clinical outcomes. In a retrospective cohort study, achieving the ideal PK/PD target in other β-lactams such as cefepime and ceftazidime demonstrated greater clinical success and bacteriological eradication; the PK/PD target established was 100% *fT_>Mic_*, being compared with a group with *fT_>MIC_* lower than 100% [40]. Another study that demonstrated better clinical outcomes related to achieving PK/PD targets was the defining antibiotic levels in intensive care unit patients (DALI) study, which was a prospective, multinational PK point-prevalence study and showed that the group that did not achieve the Pk/PD target of 50% *fT_>MIC_* were more than 30% less likely to have a positive clinical outcome (odds ratio [OR], 0.68; *p* = 0.009) [41]. This also demonstrated that increasing 50% *fT_>MIC_* and 100% *fT_>MIC_* ratios was associated with positive clinical results (OR, 1.02 and 1.56, respectively; *p* < 0.03), with significant impact on sickness severity status [41]. Another retrospective study showed a significant correlation with successful clinical outcomes related to achieving a different PK/PD target, using the steady stationary concentration divided by the minimum inhibitory concentration of the isolated bacteria (C_ss_/MIC ratio) instead of percentage of time above the MIC (OR = 12.250, 95% CI 1.268–118.361; *p* = 0.03) [42].

High-dose continuous-infusion optimized by TDM may represent a useful mechanism when a carbapenemase producing microorganisms is present [42]. However, more high-quality studies are required to approach this setting. In our research, antimicrobial resistance outcome was not studied directly, and only antimicrobial persistence was reported in two studies. Similarly, adverse reactions by systems and patient readmission did not get positive results for the quality of the selected research.

A pharmacoeconomic analysis related to TDM should include assessing the incidence of drug-induced adverse reactions, reduction in total length of hospitalization, cure rates, mortality rates, and cost savings associated with monitoring plasma levels of drugs. Unfortunately, very few articles have reviewed the pharmacoeconomic impact of this practice and, to our knowledge, there are no studies related specifically to TDM of carbapenems and cost-effectiveness. Although, for other drugs, TDM practice remains cost-effective through improved clinical outcomes, like biologic therapies in inflammatory bowel disease through reducing dosing and improving disease control [43] or digoxin through toxicity reduction [44].

Regarding antibiotics, there are few studies demonstrating significant cost reductions when TDM is performed, especially in aminoglycosides therapy. Bootman et al. performed a cost-effective analysis of burn patients, demonstrating a saving of $6689/patient in direct costs when patient’s gentamicin therapy was monitored by a clinical pharmacokinetic service [45]. Another study performed by Burton et al. found a reduction in length of stay and a potential reduction of costs when aminoglycosides TDM was put into practice [46]. Improvement of quality of patient care, reduction of patient length of stay, reduction of mortality, and earlier attainment of therapeutic drug concentrations in intervention groups compared to control groups have demonstrated potential costs savings [47]. Nonetheless, further studies are needed to demonstrate that TDM is a cost-effective practice [48].

Finally, our meta-analysis has several limitations. First, only a few RCTs met the criteria to be included in our analysis. As such, more RCTs are needed to identify true differences between groups. Second, participants and healthcare staff were aware of the group assignments in all included studies, and selective reporting and follow-up were problematic, possibly resulting in performance bias. Lastly, many of the secondary outcomes had different ways of being measured, therefore, it was not possible to do effective comparisons. It is imperative to carry out higher quality research to approach carbapenem TDM effectiveness in critically ill patients.

## 4. Materials and Methods

### 4.1. Data Sources and Searches

We performed a systematic review as per the guidelines established by the Cochrane Collaboration [49] and the Preferred Reporting Items for Systematic Review and Meta-Analysis (PRISMA) statement [41] on EMBASE, PubMed/MEDLINE, Cochrane Central Register of Controlled Trials (CENTRAL) and ClinicalTrials.gov databases updated to 15 December 2020. We used a population, intervention, comparators, and outcomes (PICO) format question [42] for the search strategy.

Search strategy associated the Medical Subject Heading (MeSH) and keywords linked to carbapenems with terms for critical illness, septic shock, sepsis, therapeutic drug monitoring, antibiotic treatment outcome, clinical outcomes, and antimicrobial drug resistance (full search strategy is in Table S1 and Table S2). Two authors (J.B. and S.L.) independently scrutinized full texts to include those who meet the selection criteria. When the two independent reviewers did not agree, a third reviewer (R.B.) made the selection.

### 4.2. Search Eligibility Criteria

We considered RCTs and comparative retrospective or prospective cohort studies as preferred study designs for inclusion. Suitable studies compared carbapenem TDM to standard care in adult critically ill or sepsis/septic shock patients. Selected research had to address at least one of the following outcomes of effectiveness determined by the authors: mortality (primary), morbidity, clinical cure, microbiological eradication, antimicrobial resistance, drug-related side effects, and adverse reactions, and achievement of target plasma concentrations (secondary). We excluded cross-sectional studies, intervention, or observational studies without a control group and research carried out in the pediatric population.

### 4.3. Data Extraction and Assessment of Methodological Quality

We collected all the papers found in a free web application Rayyan QCRI^®^, where two authors (J.B. and S.L.) separately scrutinized full texts for inclusion and remove duplicates. Three authors (Y.F., H.L., and N.D.) independently assessed randomized controlled trials (RCTs) for risk of bias adopting the Cochrane risk of bias tool [40] and observational studies applying the Newcastle–Ottawa scale (NOS) [43]. RCTs were acknowledged as having a low risk of bias (all features graded as low risk), high risk of bias (one or more items classified as high risk), or unclear risk of bias (one or more items rated as unclear risk of bias and no items within the high risk). Observational studies were classified using NOS as low risk of bias (7–9 stars), moderate risk of bias (4–5 stars), and high risk of bias (0–3 stars). Disparities were resolved by consensus with another author as required.

### 4.4. Data Synthesis

We performed a systematic and descriptive review and did a meta-analysis of those outcomes with more than one research and the same study design (RCTs and observational studies separately). We employed Mantel–Haenszel and inverse-variance, and DerSimonian and Laird models within the random-effects model, for continuous and dichotomous outcomes, respectively [44]. Merged estimates were expressed within 95% confidence intervals (CIs), as mean differences (MDs) for continuous outcomes and as relative risks (RRs) for dichotomous outcomes. A two-sided *p*-value < 0.05 was interpreted as statistically significant. To evaluate heterogeneity, we performed the Chi-square (χ^2^) test, with significance defined as *p*-value < 0.05, and the I^2^ statistic (≥50% was interpreted as severe heterogeneity) [45]. We did not assess publication bias because of the reduced number of studies identified. Analyses were done with Review Manager (RevMan version 5.4, The Nordic Cochrane Centre, The Cochrane Collaboration, Copenhagen, Denmark, 2014).

## 5. Conclusions

This systematic review and meta-analysis did not find a significant association between carbapenem TDM and favorable clinical outcomes, including mortality, reduced ICU stay, microbiological, or clinical cure, possibly because of limited evidence. Higher quality longitudinal studies are required to establish TDM-guided therapy effectiveness in the critically ill patient setting.

## Figures and Tables

**Table 1:** Description of studies selected.

Author, Year	Single or Multi-Centre/Design/N	Population/Intervention/Comparator/PK	Optimization Algorithm	Clinical Outcomes	Follow-Up Duration
A. Fournier, 2018 [26]	Prospective monocentric, randomized, controlled trial *n* = 38	Population Burn patients treated with intravenous AB. Intervention TDM of AB and dose readjustment Comparator Patients without dose adjustment based on TDM. Pharmacokinetics: Bioanalysis methodology: HPLC-MS/MS; sample: serum; result: no mean concentrations were presented in patients with carbapenems	One step adjustment: ↓ Dose: in cases of excessive minimum levels >150% of the upper limit ↑ Dose: in cases of minimum levels between 50–100% of the objective Two-step adjustment: ↓/↑ Dose: in cases of trough levels >150 or 200% of the upper limit or 10–50% variation from minimum target Adaptation to patients according to clinical history and specific population. Interruption of antibiotic treatment in case of toxicity	Primary PKs: t to achieve anti-infective serum concentrations. Serum monitoring of AB outside the target range in a single treatment cycle Secondary PK: estimation of the favorable clinical outcome (resolution of infection episodes). Monitoring of AB concentrations within the target range. Total antibiotic consumption	23 October 2013 and 31 October 2016 (3 years)
JJ. De Waele, 2013 [27]	Monocenter, prospective, partially blinded, and randomized controlled trial *n* = 41	Population Patients receiving MEM or PTZ AB. Age: ≥18 years. Normal kidney function Intervention TMD was performed daily, allowing dosage adjustment in intervals outside the objective (100% *fT_>4MIC_*). AB administration: according to an extended infusion protocol Comparator Control group: TDM was developed daily. However, the physician did not know the results. Data used for comparison only Pharmacokinetics Bioanalytical Methodology: UHPLC-MS/MS, internal standard oxacillin; sample: serum; results: median AB concentration: <2 mg/mL (before randomization), interquartile range: <2–4 mg/L MEM AB	MIC: 2 mg/L for MEM; 16mg/mL PTZ Minimum target concentration: >8 mg/L for MEM ↑ dosing frequency: If concentration is <4 MIC (1 g every 6 h for MEM) ↑ 50% dosing frequency: If concentration <4 MIC No action: 4–10 MIC ↓ 50% dosing frequency: >10_xMIC_	Primary outcome: defined target: 100% *fT_>MIC_* First 72 h: 100% *fT_>4MIC_* Baseline value and 72 h after the start of treatment: Comparison of *fT_>MIC_* and *fT_>4MIC_* (intervention and control group) Secondary Outcome: absolute values of *fT_>_*_MIC_ and *fT_>4MIC_* End of study: evaluation of clinical outcome and absence or persistence of bacteria at day 7	April 2011 and February 2012, follow up 7 days
A. Aldaz, 2020 [28]	Retrospective, unicentric cohort study *n* = 154	Population ICU patients with MEM AB treatment and dose administered according to TDM (*n* = 77) Intervention Propensity score-balanced patients receiving MEM dose-adjusted by TDM Comparator Patients with MEM AB treatment according to standard recommendations (without TDM). Dose adjustment: in patients with renal failure according to the recommendations established in the package insert Pharmacokinetics Bioanalytical methodology: HPLC; sample: serum; result: mean Cmax 27.21 µg/mL and mean Cmin: 6.69 µg/mL (TDM cohort) of MEM	n/a	Primary outcome: PCT measure with ≥80% reduction in relation to maximum levels obtained at the end of AB treatment with MEM Secondary outcome: clinical remission, microbiological remission, length of hospital stay, length of stay in ICU Side effects; hospital mortality, mortality 14 days after MEM treatment; sepsis score according to SOFA at admission and discharge	May 2011–December 2017 (67 months)
B. Meyer, 2019 [29]	Single-centre retrospective cohort study *n* = 247	Population Critically ill adult patients with administration of standard doses of MEM antibiotic (*n* = 101); critically ill adult patients with administration of MEM antibiotic dose according to MDD (*n* = 146) Intervention Individualized treatment of MEM AB guided by TDM Comparator Patients with MEM AB treatment according to standard recommendations (without TDM) Pharmacokinetics Bioassay methodology: HPLC; sample: plasma; results: 3.2 measurement of the plasma level in the TDM cohort. MEM concentrations average and per patient were not mentioned	n/a	Primary outcome: correlation of TDM and MEM AB use in critically ill patients Secondary outcome: length of stay and survival	n/a
C. Mcdonald, 2016 [30]	Retrospective, monocentric cohort study *n* = 98	Population ICU patients with administration of MEM antibiotic doses higher than those recommended (3–6 g/day) (*n* = 93 patients) (MEM *n* = 47 patients (LD = 22) (HD = 25) Intervention ↑ Doses at those recommended, when plasma free drug concentrations were below local PK/PD targets. Comparator Licensed doses usage of either MEM Pharmacokinetics Bioanalytical methodology: HPLC; sample: plasma; results: plasma MEM AB concentrations were 44 µg/mL (authorized dose group) and 81 µg/mL (high dose group)	n/a	Primary outcome measures: switch to narrower spectrum BA due to favorable outcome and resolution of infection. This was verified by microbiological data Results of the second day: healing failure, side effects by organic system, *fT_>100% MIC_* of isolated microorganisms, dose changes, duration of therapy, dosage, microbiological control, de-escalation, length of stay in the ICU, hospital destination (discharge from ICU, interhospital transfer and mortality), demographic variables	n/a

*n*: number of participants; AB: antibiotic; ↑: increase; ↓: decrease; PK: pharmacokinetics; n/a: not available; MEM: meropenem; PTZ: piperacillin/tazobactam; TDM: therapeutic drug monitoring; *fT_>MIC_*: time fraction above of the minimum inhibitory concentration; C_max_: maximum concentration; ICU: intensive care unit; C_min_: minimum concentration; UHPLC-MS/MS: ultra-high performance liquid chromatography-tandem mass spectrometry; PK/PD: pharmacokinetics/pharmacodynamics; PCT: procalcitonin; LD: licensed dose; HD: high dose; SD: standard deviation, HPLC: high-performance liquid chromatography

**Table 2:** Clinical Outcome.

Study	Timing of Outcome	TDM Cohort *n* (%)/Mean (SD)/Median (IQR)	Comparator Group *n* (%)/Mean (SD)/Median (IQR)	OR/RR/MD (95% CI)	*p-Value*	Ref
Outcome 1: mortality						
JJ. De Waele, 2013 * Single-centre, partially blinded RCT	At 7 days after treatment	In ICU: (4.8) Hospital and 28-day: (14.3)	In ICU: (20) Hospital and 28-day: (25)	n/a	In ICU: 0.18 Hospital and 28-day: 0.45	[27]
A. Aldaz, 2020 Single-centre, retrospective cohort study	In-hospital At 14 days after treatment	In-hospital: 20/77 (26) 14-day: 2/77 (2.6)	In-hospital: 20/77 (26) 14-day: 3/77 (3.9)	In-hospital mortality: n/a Mortality at 14 days after treatment: RR = 0.667; 95% CI 0.11 to 1.88	In-hospital: 1 14-day: 0.649	[28]
B. Meyer, 2019 Single-centre retrospective cohort study	In-hospital	20/146 (14)	24/101 (24)	n/a	0.042	[29]
C. Mcdonald, 2016 Single-centre retrospective cohort study	In-hospital	1/25 (4)	2/22 (9.1)	n/a	n/a	[30]
Outcome 2: ICU length of stay (days)						
A. Fournier, 2018 ** Single-centre not blinded RCT	n/a	27 (13.0–45.0)	20 (12.0–40.0)	n/a	n/a	[26]
A. Aldaz, 2020 Single-centre, retrospective cohort study	n/a	8 (3–98)	7 (3–99)	n/a	0.473	[28]
B. Meyer, 2019 Single-centre retrospective cohort study	n/a	14.7 ± 10.7	14.6 ± 9.8	n/a	n/a	[29]
C. Mcdonald, 2016 Single-centre retrospective cohort study	n/a	68.4 ± 130.8	156.6 ± 185	n/a	0.17	[30]
Outcome 3: clinical cure						
A. Fournier, 2018 ** Single-centre not blinded RCT	n/a	Meropenem: 14/19 (34.2) Imipenem-cilastatin: 1 (2.4) Ertapenem: 1 (2.4)	Meropenem: 13/19 (31.7) Imipenem-cilastatin: 1 (2.4) Ertapenem: 1 (2.4)	n/a	n/a	[26]
A. Aldaz, 2020 Single-centre, retrospective cohort study	May 2011–December 2017 (67 months)	Reduction 80% in PCT: 55/77 (71.43)	Reduction 80% in PCT: 41/77 (53.25)	n/a	0.02	[28]
C. Mcdonald, 2016 Single-centre retrospective cohort study	n/a	Cessation or de-escalation of antibiotic 21/25 (84)	15/22 (68.18)	n/a	n/a	[30]
Outcome 4: microbiological eradication						
A. Aldaz, 2020 Single-centre, retrospective cohort study	May 2011–December 2017 (67 months)	66/77 (85.7)	62/77 (80.5)	n/a	0.39	[28]
C. Mcdonald, 2016 Single-centre retrospective cohort study	n/a	20/22 (80)	13/22 (59.1)	n/a	0.48	[30]
Outcome 5: target attainment						
A. Fournier, 2018 ** Single-centre not blinded RCT	n/a	C_min_ value: 28/36 (77,8)	C_min_ value: 15/27 (55,6)	n/a	n/a	[26]
JJ. De Waele, 2013 * Single-centre, partially blinded RCT	72 h	100% *fT_>MIC_*: (94.7%) 100% *fT_>4MIC_*: (57.9%)	100% *fT_>MIC_*: (68.4%) 100% *fT_>4MIC_*: (15.8%)	n/a	100% *fT_>MIC_*: 0.045 100% *fT_>4MIC_*: 0.007	[27]
C. Mcdonald, 2016 Single-centre retrospective cohort study	n/a	100% *fT_>MIC_*: 15/28 (53.6)	100% *fT_>MIC_*: 10/22 (45.5)	n/a	0.57	[30]
Outcome 6: antimicrobial resistance						
JJ. De Waele, 2013 *^,¥^ Single-centre, partially blinded RCT	7 days	1 (n/a)	5 (n/a)	n/a	0.09	[27]
A. Aldaz, 2020 ^¥^ Single-centre, retrospective cohort study	May 2011–December 2017 (67 months)	11/77 (14.3)	15/77 (19.5)	n/a	0.39	[28]
Outcome 7: adverse reactions						
A. Aldaz, 2020 Single-centre, retrospective cohort study	May 2011–December 2017 (67 months)	Gastrointestinal: 11/77 (14.29) Hematologic: 40/77 (51.95) CNS: 4/77 (5.19) Dermatological: 3/77 (3.90) Hepatobiliary: 36/77 (46.75)	Gastrointestinal: 11/77 (14.29) Hematologic: 31/77 (40.26) CNS: 10/77 (12.99) Dermatological: 2/77 (2.60) Hepatobiliary: 36/77 (46.75)	n/a	Gastrointestinal: 1 Hematologic: 0.148 CNS: 0.093 Dermatoogical: 0.649 Hepatobiliary: 1	[28]
C. Mcdonald, 2016 Single-centre retrospective cohort study	n/a	Hepatic Toxicity: Hepatocellular derangement: 5/28 (17.9) Cholestasis: 7/28 (28.0) Hematological Toxicity: Thromocytopenia: 3/28 (10.7) Neutropenia: 1/28 (3.6) Need for CRRT Incidence: 0/28 Resolved: 0/28	Hepatic Toxicity: Hepatocellular derangement: 7/22 (31,8) Cholestasis: 3/22 (13.6) Hematological Toxicity: Thromocytopenia: 2/22 (9.1) Neutropenia:1/22 (4.5) Need for CRRT Incidence: 2/22 (9.1) Resolved: 1/22 (4.5)	n/a	Hepatic Toxicity: Hepatocellular derangement: 0.25 Cholestasis: 0.32 Hematological Toxicity: Thromocytopenia: 0.85 Neutropenia: 0.95 Need for CRRT Incidence: 0.10 Resolved: 0.25	[30]
Outcome 8: hospital readmission						
A. Aldaz, 2020 Single-centre, retrospective cohort study	May 2011–December 2017 (67 months)	5/77 (6.49)	7/77 (9.09)	n/a	0.548	[28]

*n*: number of participants; SD: standard deviation; IQR: interquartile range; OR: odds ratio; RR: relative risk; MD: mean difference; RCT: randomized controlled trial; ICU: intensive unit care; n/a: not available; PCT: procalcitonin; C_min_: minimum plasma concentration; *fT*_>MIC_: time fraction above minimum inhibitory concentration; CNS: central nervous system; CRRT: continuous replacement renal therapy. All studies evaluated Meropenem. * The results are not discriminated by type of antibiotic (meropenem and piperacillin/tazobactam together)/absolute frequencies are not specified. ** Meropenem e Imipenem. ^¥^ Microbiological persistence.

**Table 3:** Risk of bias in observational studies according Newcastle-Ottawa scale (NOS) tool.

Study	Representativeness of Exposed Cohort	Selection of Non-Exposed Cohort	Ascertainment of Exposure and Blinding	Outcome not Present at Outset	Study Controls for Important Confounder ± Additional Confounders, Including Differences in Care	Blind Assessment of Outcome	Follow-Up Long Enough	Follow-Up Adequacy	Total Number of Stars (Out of 9)
	Selection				Comparability	Outcome			
A. Aldaz, 2020	★	★		★	★★		★	★	7
B. Meyer, 2019	★	★		★			★		4
C. Mcdonald, 2016	★	★			★		★		4

A study can be awarded a maximum of one star (★) for each item except comparability, for which a study can be awarded a maximum of two stars (★★).

## Supplementary Materials

The following are available online at https://www.mdpi.com/2079-6382/10/2/177/s1. Table S1: Search Strategy PROSPERO, Table S2: PROSPERO Protocol.
