# Supplementary material for: Carbapenem Therapeutic Drug Monitoring in Critically Ill Adult Patients and Clinical Outcomes: A Systematic Review with Meta-Analysis
Source: Antibiotics (Basel). 2021 Feb 10;10(2):177. doi: 10.3390/antibiotics10020177 (PMC7916352; doi:10.3390/antibiotics10020177)
Supplement: Supplementary file 1 [file antibiotics-10-00177-s001.zip › Supplementary material_1/T1. Supplementary. Search Strategy PROSPERO.pdf]

Table 1S. Search strategy

Review question
What is the clinical effectiveness of carbapenem therapeutic drug monitoring (TDM) in the critically ill patient?

PICO format
Population: Patients with sepsis, septic shock o critically ill treated with carbapenems
Intervention: Therapeutic drug monitoring
Comparator: Not performing therapeutic drug monitoring
Outcomes: Clinical Effectiveness (Antimicrobial drug resistance; antibiotic resistance; Mortality; Morbidity; Treatment Outcome; Length of Stay; Hospitalization; Patient Readmission; Drug-Related Side Effects and Adverse Reactions; Pharmacokinetics; clinical cure; microbiological eradication; target plasma concentration)
Study design: Any type of study design

Filters
Adult: 19+ years; Humans

Data Bases
<u>Pubmed/Medline</u>
<u>Embase</u>
<u>ClinicalTrials.gov</u>
<u>Cochrane Central Register of Controlled Trials (CENTRAL)</u>

Key words
Critical illness, septic shock, sepsis, carbapenems, drug monitoring, treatment outcome

Terms
<u>Patients with sepsis, septic shock o critically ill treated with carbapenems</u>

- Care, critical
- Critical illness
- Septic shock
- Sepsis
- Carbapenems
- Panipenem
- Ertapenem
- Meropenem
- Meropenem and vaborbactam
- Doripenem
- Imipenem
- Cilastatin Imipenem Drug Combination
- Imipenem, cilastatin and relebactam
- Sanfetrinem
- Sanfetrinem cilexetil

<u>Therapeutic drug monitoring</u>

- Drug Monitoring
- Therapeutic drug monitoring

<u>Clinical Effectiveness</u>

- Antimicrobial drug resistance
- Antibiotic resistance
- Mortality
- Morbidity

• Treatment Outcome<br>• Length of Stay<br>• Hospitalization<br>• Patient Readmission<br>• Drug-Related Side Effects and Adverse Reactions<br>• Pharmacokinetics<br>• Clinical cure<br>• Microbiological eradication<br>• Target plasma concentration
**Search algorithm**<br>1. Care, critical<br>2. Critical illness<br>3. Septic shock<br>4. Sepsis<br>5. 1 OR 2 OR 3 OR 4<br>6. Carbapenems<br>7. Panipenem<br>8. Ertapenem<br>9. Meropenem<br>10. Meropenem and vaborbactam<br>11. Doripenem<br>12. Imipenem<br>13. Cilastatin Imipenem Drug Combination<br>14. Imipenem, cilastatin and relebactam<br>15. Sanfetrinem<br>16. Sanfetrinem cilexetil<br>17. 6 OR 7 OR 8 OR 9 OR 10 OR 11 OR 12 OR 13 OR 14 OR 15 OR 16<br>18. 5 AND 17<br>19. Drug Monitoring<br>20. Therapeutic drug monitoring<br>21. 19 OR 20<br>22. Antimicrobial drug resistance<br>23. Antibiotic resistance<br>24. Mortality<br>25. Morbidity<br>26. Treatment Outcome<br>27. Length of Stay<br>28. Hospitalization<br>29. Patient Readmission<br>30. Drug-Related Side Effects and Adverse Reactions<br>31. Pharmacokinetics<br>32. Clinical cure<br>33. Microbiological eradication<br>34. Target plasma concentration<br>35. 22 OR 23 OR 24 OR 25 OR 26 OR 27 OR 28 OR 29 OR 30 OR 31 OR 32 OR 33 OR 34<br>36. 18 AND 21 AND 35<br>37. Humans<br>38. Adult: 19+ years<br>39. 36 AND 37 AND 38<br>40. Apply limits if applicable

Search strategy for Embase
('critically ill patient'/exp OR 'critical illness'/exp OR 'sepsis'/exp OR 'septic shock'/exp) AND ('carbapenem'/exp OR 'ertapenem'/exp OR 'meropenem'/exp OR 'meropenem plus vaborbactam'/exp OR 'imipenem'/exp OR 'cilastatin plus imipenem'/exp OR 'doripenem'/exp OR 'cilastatin sodium plus imipenem plus relebactam'/exp OR 'sanfetrinem'/exp OR 'sanfetrinem cilexetil'/exp OR 'panipenem'/exp) AND ('drug monitoring'/exp OR 'therapeutic drug monitoring') AND ('antibiotic resistance'/exp OR 'mortality'/exp OR 'morbidity'/exp OR 'treatment outcome'/exp OR 'hospitalization'/exp OR 'hospital readmission'/exp OR 'length of stay'/exp OR 'side effect'/exp OR 'pharmacokinetics'/exp OR 'cure rate'/exp OR 'microbiological eradication' OR 'target plasma concentration')

Search strategy for Pubmed/Medline
((((((care, critical[MeSH Terms]) OR (critical illness[MeSH Terms])) OR (septic shock[MeSH Terms])) OR (sepsis[MeSH Terms])) AND ((((("Carbapenems"[Mesh] OR "panipenem" [Supplementary Concept]) OR "Ertapenem"[Majr]) OR "Doripenem"[Majr]) OR ("Meropenem"[Mesh] OR "meropenem and vaborbactam" [Supplementary Concept])) OR ("Imipenem"[Mesh] OR "Cilastatin, Imipenem Drug Combination"[Mesh] OR "imipenem, cilastatin and relebactam" [Supplementary Concept])) OR ("sanfetrinem" [Supplementary Concept] OR "sanfetrinem cilexetil" [Supplementary Concept]))) AND ((("Drug Monitoring"[Mesh]) OR (therapeutic drug monitoring))) AND ((antimicrobial drug resistance[MeSH Terms]) OR ((((((((("Mortality"[Mesh]) OR "Morbidity"[Mesh]) OR "Treatment Outcome"[Mesh]) OR "Length of Stay"[Mesh]) OR "Hospitalization"[Mesh]) OR "Patient Readmission"[Mesh])) OR "Drug-Related Side Effects and Adverse Reactions"[Mesh]) OR "Pharmacokinetics"[Mesh]) OR (((clinical cure) OR (microbiological eradication)) OR (target plasma concentration))))

Search strategy for ClinicalTrials.gov
Critical Illness | drug monitoring | Adult, Older Adult

Search strategy for CENTRAL
"carbapenems" OR "Ertapenem" OR "Meropenem" OR "Imipenem" OR "Doripenem" OR "Panipenem" OR "Sanfetrinem" in Title Abstract Keyword AND "drug monitoring" in Title Abstract Keyword

Eligibility criteria
Inclusion criteria

- Eligible studies compared carbapenem TDM to empiric care in adult critically ill or sepsis/septic shock patients.
- Randomized controlled trials (RCTs)
- Comparative prospective or retrospective cohort studies
- Included studies had to report an effect estimate for at least one of the following outcomes of effectiveness determined by the authors: mortality, morbidity, clinical cure, microbiological eradication, antimicrobial resistance, drug-related side effects and adverse reactions and achievement of target plasma concentrations.

Exclusion criteria

- Cross sectional studies
- Intervention or observational studies without control group
- Research carried out in pediatric population
