# Supplementary material for: Carbapenem Therapeutic Drug Monitoring in Critically Ill Adult Patients and Clinical Outcomes: A Systematic Review with Meta-Analysis
Source: Antibiotics (Basel). 2021 Feb 10;10(2):177. doi: 10.3390/antibiotics10020177 (PMC7916352; doi:10.3390/antibiotics10020177)
Supplement: Supplementary file 1 [file antibiotics-10-00177-s001.zip › Supplementary material_1/T2. Supplementary. PROSPERO Protocol.pdf]

Table 2S. PROSPERO Protocol

**Title of the review**	Carbapanem therapeutic drug monitoring in critically ill patient as a strategy to decrease antimicrobial resistance: a systematic review
**First reviewer**	Dr. Rosa Helena Bustos Cruz
**Team of reviewers**	Dr Sharon Lechtig Wasserman<br>Dr Jhósep Andrés Blanco Mejía<br>Dr Yuli Viviana Fuentes Barreiro<br>Dr Hans Nicolás Liebich Rey<br>Dr Nicolás Díaz Pinilla
**Supervisor/Project PI**	Dr Rosa Helena Bustos Cruz
**Clinical Portfolio Group**	Dr Sharon Lechtig Wasserman<br>Dr Jhósep Andrés Blanco Mejía<br>Dr Hans Nicolás Liebich Rey<br>Dr Nicolás Díaz Pinilla – Physician, Researcher assistant, University of La Sabana<br>Dr Yuli Viviana Fuentes Barreiro – Master in epidemiology, University of La Sabana<br>Dr Rosa Helena Bustos Cruz – Research director, TDM group, University of La Sabana; Dr Eberhard Karls, Tübingen Universität
**Project title**	Standardization of a methodology for the quantification of antimicrobials used for the treatment of multi-resistant bacteria through the use of two types of biosensors and production of anti-antimicrobial antibodies.

**Support**	
**SR overview**	Advice sought from Dr. Bustos
**Protocol development**	Training obtained from research director Dr Bustos, Dr Fuentes (who has a MSc in epidemiology) and also experience obtained from research done during medical school.
**Literature searching**	Advice from Dr. Bustos
**Quality appraisal**	On proccess
**Data Extraction**	Not started
**Synthesis**	Not started
**Writing up**	Not started

**1. Background to review**
Therapeutic drug monitoring (TDM) is defined by the clinical practice of measuring specific drugs at designated intervals to maintain a constant concentration in a patient's bloodstream, thereby optimizing individual dosage regimens (Kang et al, 2009). The goal of this process is to individualize therapeutic regimens for optimal patient benefit, based on pharmacokinetic and pharmacodynamic parameters (Kang et al, 2009). TDM plays an important role in the development of safe and effective therapeutic medications and individualization of these drugs (Mandal et al,

2019). Indications of measuring drug concentrations include monitoring compliance, individualizing treatment during early therapy and dosage changes, diagnosing undertreatment, avoiding toxicity, monitoring drug interactions, and guiding withdrawal of therapy (Mandal et al, 2019).

Accumulating evidence stresses the importance of maintaining free drug concentrations that exceed the bacterial minimum inhibitory concentration for a specified time to prevent toxicity, antibiotic resistance, or treatment failure (Neugebauer et al, 2019). It has been demonstrated that therapeutic drug monitoring is useful to reach adequate therapeutic ranges in certain drugs, especially for those with a narrow therapeutic window, drugs with marked pharmacokinetic variability, medications for which target concentrations are difficult to monitor, and drugs known to cause severe adverse effects (10.3904/kjim.2009.24.1.1 Junaid et al, 2019). It has been also proved that there exists intra and inter-individual pharmacokynetic variability between patients, especially the critically ill (Muller et al, 2018).

Carbapenems are antibiotics endowed with a broader spectrum and greater resistance to β-lactamases than other β-lactams. Due to their qualities, these antibiotics are crucial in empirical therapy, in the monotherapy of severe hospital-acquired infections -and even that of some community-acquired infections- as well as in the directed therapy of infections due to multiresistant Gram-negative bacteria (Fresnadillo et al, 2010). Carbapenems are bactericidal although their activities vary and can be compromised by bacterial resistance mechanisms (Breilh et al, 2013). Due to extend-spectrum beta-lactamases (ESBLs) producing organisms and the increasing resistance challenges represented by beta-lactamases or carbapenemases (e.g. KPC or NDM), carbapenems still assume a great role in the treatment of serious infections (Breilh et al, 2013).

Many pharmacokinetic changes may be observed for carbapenems in intensive care unit (ICU) patients, such as increased volume of distribution, modified antibiotic clearance depending on renal or hepatic function, modified protein binding caused by hypoalbuminaemia or modified tissue penetration (Blot et al, 2014; Van Harten, 2012; Pea et al, 2012). Since these differences may have implications for the clinical efficacy and the correct dosage of antimicrobial agents, some scarce studies aimed at determining the optimal antibiotic regimen for ICU patients (De Waele, 2013). However, it is not clear if the TDM practice in carbapenems is related with better clinical outcomes in the critically ill patients.

The research to be undertaken will help to build on this as yet inconclusive evidence to elucidate the relationship between performing carbapenem TDM in critically ill patients and clinical outcomes such as reducing mortality, morbidity, hospital length of stay and readmission. Findings will inform the development of strategies for reducing negative outcoomes in this patient group that could be tested in a subsequent research proposal.

Aim

To identify the efficacy of TDM of carbapenems in the critically ill patient in terms of clinical outcomes as a strategy to decrease antimicrobial resistance. The understanding may allow potentially effective interventions for improving antibiotic therapy in the clinical setting and to reduce antimicrobial resistance, especially for gram negative bacteria, to be designed and later systematically evaluated in more in-depth studies

2. Specific objectives
1. To clarify the evidence base available around the relationships between performing therapeutic drug monitoring on carbapenems and reducing mortality in critically ill patients or with severe sepsis/septic shock. Clarification will be made by a systematic review of the evidence base of journals and abstracts in this topic area, looking at clinical trials, case control studies and cohort studies.<br><br>2. To clarify the evidence base available around the relationships between performing therapeutic drug monitoring on carbapenems and reducing morbidity in critically ill patients or with severe sepsis/septic shock. Clarification will be made by a systematic review of the evidence base of journals and abstracts in this topic area, looking at clinical trials, case control studies and cohort studies.<br><br>3. To clarify the evidence base available around the relationships between performing therapeutic drug monitoring on carbapenems and reducing hospital length of stay in critically ill patients or with severe sepsis/septic shock. Clarification will be made by a systematic review of the evidence base of journals and abstracts in this topic area, looking at clinical trials, case control studies and cohort studies.<br><br>4. To clarify the evidence base available around the relationships between performing therapeutic drug monitoring on carbapenems and achieving Microbiological eradication in critically ill patients or with severe sepsis/septic shock. Clarification will be made by a systematic review of the evidence base of journals and abstracts in this topic area, looking at clinical trials, case control studies and cohort studies.<br><br>5. To clarify the evidence base available around the relationships between performing therapeutic drug monitoring on carbapenems and reducing Antimicrobial resistance in critically ill patients or with severe sepsis/septic shock. Clarification will be made by a systematic review of the evidence base of journals and abstracts in this topic area, looking at clinical trials, case control studies and cohort studies.<br><br>6. To clarify the evidence base available around the relationships between performing therapeutic drug monitoring on carbapenems and reducing drug related side effects or adverse reactions in critically ill patients or with severe sepsis/septic shock. Clarification will be made by a systematic review of the evidence base of journals and abstracts in this topic area, looking at clinical trials, case control studies and cohort studies<br><br>7. To clarify the evidence base available around the relationships between performing therapeutic drug monitoring on carbapenems and achievement of targeted plasma concentration in critically ill patients or with severe sepsis/septic shock. Clarification will be made by a systematic review of the evidence base of journals and abstracts in this topic area, looking at clinical trials, case control studies and cohort studies<br><br>8. To identify any other factors in these patients that are thought to also be involved in their admission. Along with the co-morbidities of anxiety and depression. These other factors include ability to cope and self-manage their condition and also other co morbidities and social factors that may affect their ability to cope or self-manage. This cannot be more specific until an examination of the evidence is done

3. a) Criteria for including studies in the review	
**i. Population, or participants and conditions of interest**	Patients with sepsis, septic shock or critically ill treated with carbapenems
**ii. Interventions or exposures**	Performing Therapeutic drug monitoring in clinical setting
**iii. Comparisons or control groups**	Not performing therapeutic drug monitoring
**iv. Outcomes of interest**	Clinical Effectiveness (Antimicrobial drug resistance; antibiotic resistance; Mortality; Morbidity; Treatment Outcome; Length of Stay; Hospitalization; Patient Readmission; Drug-Related Side Effects and Adverse Reactions; Pharmacokinetics; clinical cure; microbiological eradication; target plasma concentration)
**v. Setting**	Hospital
**vi. Study designs**	*Case-control/cohort studies, RCT*
**3. b) Criteria for excluding studies not covered in inclusion criteria**	
• Cross sectional studies, observational or intervention studies without control group<br>• Studies that include pediatric patients<br>• Studies that don't relate with clinical outcomes mentioned above	

4. Search methods	
**Electronic databases**	PUBMED/MEDLINE<br>COCHRANE (CENTRAL)<br>EMBASE<br>ClinicalTrials.gov
**Other methods used for identifying relevant research**	Identifying possible data from conferences attended
**Journals hand searched**	Not applicable

5. Methods of review	
**Details of methods**	A systematic literature review was performed in accordance with the guidelines established by the Cochrane Collaboration and the PRISMA statement in PubMed/MEDLINE, EMBASE, Cochrane Central Register of Controlled Trials (CENTRAL) and ClinicalTrials.gov databases to 17 December 2020. The search strategy was based on the Population, Interventions,Comparators and Outcomes (PICO) format question. Two authors (JB and SL) independently reviewed full texts for inclusion. A third reviewer (RB) was consulted when the two independent reviewers disagreed.

**Quality assessment**	Protocol will define the method of literature critique/ appraisal use, and will use<br>STROBE tool for relevant content and methodology used in the each of the<br>papers to be reviewed.
**Data extraction**	All the papers found were collected in RAYYAN® where two authors (JB and SL) independently reviewed full texts for inclusion; duplicates were removed.<br>Endnote X9 to be used to keep track of references.<br>Reviewer number 1 (SL) will review first, followed by reviewer number 2 (JB),<br>which will be done independently. If necessary, reviewer number 3 and 4 will review if there are any disparities between the two reviewers.
**Risk of bias**	Three authors (YF, HL and ND) independently assessed RCTs for risk of bias using the Cochrane risk of bias tool and non-randomized studies using the Newcastle-Ottawa scale (NOS). RCTs were considered at low risk of bias if all items were rated low risk of bias, at high risk of bias if one or more items were rated high risk of bias, and at unclear risk if one or more items were rated unclear risk of bias and no items were rated high risk of bias. Non-randomized studies were considered at low risk of bias if they received 7–9 stars on the NOS (maximum 9), at moderate risk of bias if they received 4–5 stars, and at high risk of bias if they received 0–3 stars. In all cases, discrepancies were resolved by consensus with third party input as needed.
**Narrative synthesis**	Narrative synthesis will be done alongside any meta-analysis and will be carried out using a framework which consists of four elements:<br>1. Developing a theory of how the intervention works, why and for whom<br>2. Developing a preliminary synthesis of findings of included studies<br>3. Exploring relationships within and between studies<br>4. Assessing the robustness of the synthesis
**Meta-analysis**	We anticipate a relatively small number of studies and therefore primarily aimed to perform a systematic review. Outcomes for which more than one study are available will be

	included in a meta-analysis. Effect estimates will be pooled separately for RCTs and observational studies. Random-effects model, as per DerSimonian and Laird, and the Mantel-Haenszel and inverse-variance methods for dichotomous and continuous outcomes will be used, respectively (DerSimonian and Laird, 2003). Pooled estimates will be expressed as odds ratios (ORs) or relative risks (RRs) for dichotomous outcomes and as mean differences (MDs) for continuous outcomes, with 95% confidence intervals (CIs). To evaluate heterogeneity will be performed the Chi-square test, with significance defined as $p < 0.05$, and the I2 statistic (≥50% was interpreted as severe heterogeneity) (Higgins et al, 2003).<br><br>Publication bias will be assessed with a funnel plot if more than 10 studies are identified. Analyses will be done with Review Manager (RevMan version 5.3, Copenhagen: The Nordic Cochrane Centre, The Cochrane Collaboration, 2014). A two-sided p-value < 0.05 was considered statistically significant
**Grading evidence**	Not applicable

**6. Presentation of results**	
**Additional material**	Flow chart of whole process<br>Protocol<br>Data extraction form and tables<br>Forest plots of studies included in the final review
**Outputs from review**	X1 paper in high quality infectious diseases journal (antibiotics)<br>Conference presentations

**7. Timeline for review**	
**Protocol**	1 month
**Literature searching**	2 weeks
**Quality appraisal**	2 weeks
**Data extraction**	1 month
**Synthesis**	2 weeks
**Writing up**	1 month
